# Phylodynamic Analysis Complements Partner Services by Identifying Acute and Unreported HIV Transmission

**DOI:** 10.3390/v12020145

**Published:** 2020-01-27

**Authors:** Ellsworth M. Campbell, Anne Patala, Anupama Shankar, Jin-Fen Li, Jeffrey A. Johnson, Emily Westheimer, Cynthia L. Gay, Stephanie E. Cohen, William M. Switzer, Philip J. Peters

**Affiliations:** 1 Centers for Disease Control and Prevention, Atlanta, GA 30322, USA; uxf5@cdc.gov (A.P.); ikb6@cdc.gov (A.S.); jfl2@cdc.gov (J.-F.L.); jlj6@cdc.gov (J.A.J.); bis3@cdc.gov (W.M.S.); ewe9@cdc.gov (P.J.P.); 2 ICF International, Atlanta, GA 30329, USA; 3 New York City Department of Health and Mental Hygiene, New York, NY 10013, USA; ewestheimer@gmail.com; 4 Gillings School of Global Public Health, University of North Carolina at Chapel Hill, Chapel Hill, NC 27599, USA; cynthia_gay@med.unc.edu; 5 San Francisco Department of Public Health, San Francisco, CA 94102, USA; stephanie.cohen@sfdph.org

**Keywords:** phylodynamics, HIV transmission, sexual network, risk network, contact network, genetic network, acute HIV infection, MicrobeTrace, network visualization

## Abstract

Tailoring public health responses to growing HIV transmission clusters depends on accurately mapping the risk network through which it spreads and identifying acute infections that represent the leading edge of cluster growth. HIV transmission links, especially those involving persons with acute HIV infection (AHI), can be difficult to uncover, or confirm during partner services investigations. We integrated molecular, epidemiologic, serologic and behavioral data to infer and evaluate transmission linkages between participants of a prospective study of AHI conducted in North Carolina, New York City and San Francisco from 2011–2013. Among the 547 participants with newly diagnosed HIV with polymerase sequences, 465 sex partners were reported, of whom only 35 (7.5%) had HIV sequences. Among these 35 contacts, 23 (65.7%) links were genetically supported and 12 (34.3%) were not. Only five links were reported between participants with AHI but none were genetically supported. In contrast, phylodynamic inference identified 102 unreported transmission links, including 12 between persons with AHI. Importantly, all putative transmission links between persons with AHI were found among large clusters with more than five members. Taken together, the presence of putative links between acute participants who did not name each other as contacts that are found only among large clusters underscores the potential for unobserved or undiagnosed intermediaries. Phylodynamics identified many more links than partner services alone and, if routinely and rapidly integrated, can illuminate transmission patterns not readily captured by partner services investigations.

## 1. Introduction

Persons with acute HIV infection (AHI) represent the leading edge of HIV transmission because they are unaware of their infection status, are likely to continue to engage in high-risk behavior, and have characteristically high viral loads at high risk of transmission [1,2]. The leading edge of HIV transmission is also where prevention and treatment interventions are most effective [3]. Upon identification of a person with AHI, health departments can offer partner services, which can include an array of medical, preventative and psychological counseling services for persons with diagnosed HIV infection and their high-risk contacts. Confidential notification of high-risk contacts is a critical public health method used to connect those at highest risk of infection to pre-exposure prophylaxis (PrEP) and other prevention services [4], to diagnose HIV among those unaware of their infection [5,6], to mitigate high-risk behaviors [7] and to engage persons with previously diagnosed HIV in medical care [8].

Partner services investigations can be guided by social network and venue data that are collected during confidential interviews with persons with newly diagnosed HIV and their partners. These data depend on a person’s recall and their willingness to provide a partner’s private information to a public health agency, and are often incomplete, vague or anonymous [9]. Phylodynamics seeks to integrate molecular, epidemiologic (epi), immunologic, behavioral and other disparate data to offer a more holistic understanding of how multiple complex processes interact to shape transmission dynamics [10]. This holistic approach, combining partner services and phylodynamics analysis, enables identification of potential hidden transmission links and confirmation of reported transmission links where traditional epidemiologic information might be unclear, unreliable or altogether unavailable [11,12]. This complementary approach to partner services offers a more detailed understanding of pathogen transmission that can guide public health prevention and intervention efforts that are tailored to rapidly growing clusters of HIV transmission under investigation.

In this report, we integrate the complementary high-risk sexual contact and HIV genetic distance networks and conduct phylodynamics analyses to characterize transmission clusters yielded from partner services investigations.

## 2. Materials and Methods 

Screening Targeted Populations to Interrupt Ongoing Chains of HIV Transmission with Enhanced Partner Notification (STOP) was a prospective study from 2011–2013 that evaluated testing methods for the diagnosis of AHI. All participants with newly diagnosed HIV infection were offered enhanced partner services at 12 HIV testing sites across three jurisdictions: North Carolina, New York City and San Francisco (NC, NYC and SF, respectively) [13]. Details regarding participant recruitment (*n* = 86,836), inclusion criteria, standardized questionnaire and testing procedures have been previously reported [14]. Local institutional review board approvals were obtained, as indicated by local policies, for the University of California at San Francisco, the University of North Carolina at Chapel Hill and the New York City Department of Health and Mental Hygiene. A research determination in accordance with federal human participant protection regulations and Centers for Disease Control and Prevention (CDC) policies and procedures were obtained and CDC review determined that CDC was not engaged in human subject research. All study methods were carried out according to these guidelines and all data were collected with informed consent under the original study [14]. Fingerstick specimens from participants were initially screened with a point-of-care rapid HIV test (OraQuick ADVANCE Rapid HIV-1/2 Antibody Test, OraSure Technologies or Clearview HIV 1/2 STAT-PAK assay, Alere). Persons with a negative rapid test result were tested for AHI with an HIV Antigen (Ag)/Antibody (Ab) combination test (Abbott Architect HIV Ag/Ab Combo Assay; Abbott Diagnostics) and with pooled HIV-1 RNA testing using a freshly collected blood specimen. Individual specimens from a pooled specimen with positive RNA results were further tested with either the Aptima HIV-1 RNA qualitative assay (Gen-Probe), a qualitative method with a lower limit of detection of approximately 30 copies/mL, or the Abbott m2000 RealTime HIV-1 quantitative assay (Abbott Diagnostics), a quantitative method with a lower limit of quantification of 40 copies/mL, to identify the specific positive specimen(s). AHI was defined by a negative rapid HIV test result followed by a reactive HIV Ag/Ab combination assay result, or detectable HIV RNA on pooled HIV RNA testing confirmed with an individual HIV RNA test. Established HIV infection was defined as infection detected by a rapid HIV test and confirmed by a reactive Multispot HIV-1/HIV-2 Rapid Test (BioRad) or positive HIV-1 Western blot result, with discordant confirmatory results resolved with HIV-1 RNA testing. All participants were offered partner notification services and contact information was elicited for sex partners who were offered HIV testing and a standardized questionnaire. Our current analysis focuses solely on HIV-infected participants and their HIV-infected partners, with a sub-analysis on those with an available HIV polymerase (*pol*) sequence. All partners with previously diagnosed HIV infection were designated as having established infection but were not included as study participants and therefore did not provide a sample for sequence analysis.

We used the Sanger method to obtain partial HIV (*pol*) sequences from persons with available blood specimens, which were analyzed for drug resistance (DR) markers and subtype using SIERRA [15] and COMET [16], respectively. MicrobeTrace [17], a bioinformatics tool used to infer genetic networks and integrate those with contact networks, was used to determine pairwise *pol* genetic distances (*d*) according to the Tamura-Nei substitution model (TN93) [18] after pairwise sequence alignment to the HIV-1_HXB2 reference sequence (GenBank accession K03455, nucleotide positions: 2253–3869). MicrobeTrace is designed to replicate methods employed by the HIV-TRACE algorithm [19]. Transmission linkages were inferred when the genetic distance between any pair of *pol* sequences was below a threshold (*d* ≤ 1.5%) [20]. These methods have previously shown equivalent efficacy for identification of transmission pairs and yielding actionable information about variability in transmission rates among clusters [12,21,22,23]. Graphically, transmission linkages are represented by lines drawn between both nodes, where each node represents a participant’s *pol* sequence. If a participant’s *pol* sequence was linked to another according to this threshold model, both participants were labeled as clustered. Those participants whose *pol* sequence did not link to any other participant were labeled unclustered.

We used SAS (SAS Institute) version 9.4 to perform bivariate analyses using Pearson’s chi-square test and to calculate odds ratios (ORs) and 95% confidence intervals (95% CI) that examined associations between clustering status and participants’ behavioral and demographic characteristics. Drug-resistance associated mutation (DRAM) codons were included in this analysis as previous studies have demonstrated that these resistance codons have a negligible effect on network characteristics [12,20]. For each molecular link involving DRAMs, we used the symmetry of DRAM presence to differentiate transmitted mutations from de novo mutations.

**Figure 1:**
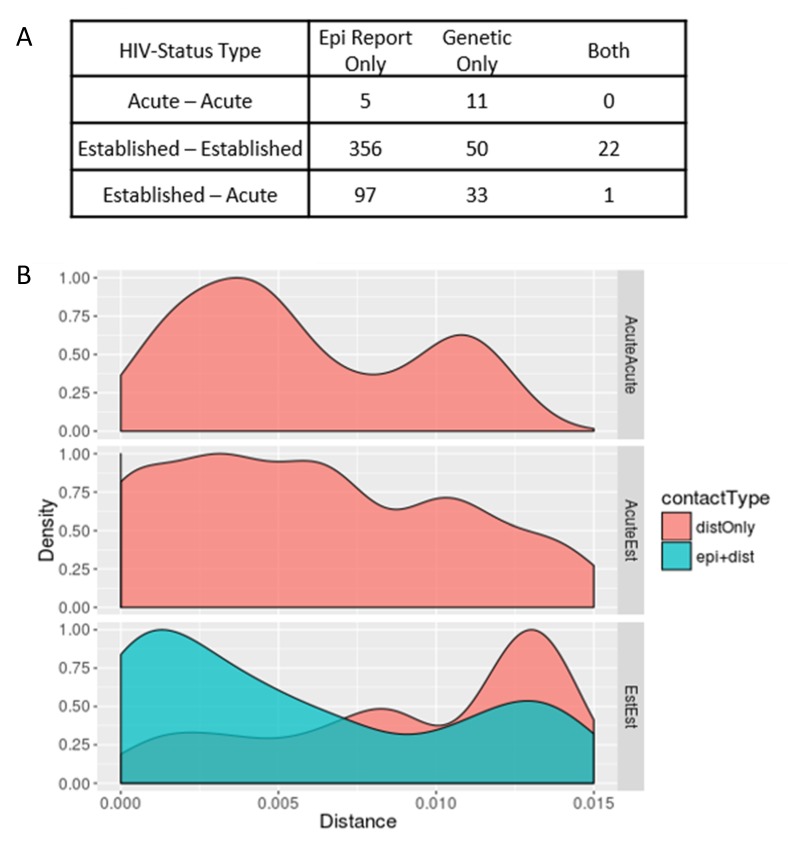
Contact and close genetic links, categorized by recency of HIV infection and type of reported contact. Links between participants were categorized according to the recency of infection, i.e., Acute-to-Acute (AcuteAcute), Acute-to-Established (AcuteEst) or Established-to-Established (EstEst). Links were also categorized by contact type, indicating whether a link was reported, genetically inferred (distOnly) or both (epi + dist). (**A**) Represents the counts of each category of link that was observed. (**B**) Kernel density plots of the pairwise genetic distances (*x*-axis), broken out by recency classification and colored by contact type (distOnly or epi + dist). Kernel density plots are smoothed and normalized functions designed to capture the density of observations at an arbitrary value, enabling comparison of disparate distributions, similar to a histogram.

HIV genetic distance and participant sexual networks were constructed independently and then integrated into a multi-partite network. Nodes represent participants and links between them were categorized as genetic only, epi only, or both (Figure 1A). Epi only links are those that were reported by one or both participants, but uncorroborated by molecular data. Note that epi only links can consist of two categories: (1) *confirmed* (*d* > 1.5%) when a *pol* sequence is available for both participants and they are sufficiently distant to rule out recent transmission or (2) *unconfirmed* when either partner’s *pol* sequence is unavailable. Each of these links were further classified by the demographic, serological and behavioral properties of the participants that they connect. For example, we calculated the absolute differences in ages, number of claimed partners, number of named partners, number of anonymous partners and whether a corresponding epi link was reported. We also categorized links according to the HIV infection status of both participants (e.g., Acute-Acute, Established-Acute, Established-Established: Figure 1A).

Topological properties of connected components (clusters) were assessed in the HIV genetic distance network, participant sexual network, and integrated networks containing both genetic and sexual networks. Each cluster was evaluated with respect to the prevalence of AHI in addition to age and geographic distributions of participants. A binomial logistic regression model was trained to predict whether a participant’s HIV *pol* sequence clustered according to demographic characteristics such as age, race/ethnicity, the number of named and anonymous high-risk partners and whether they met partners online. A random forest decision tree was used to highlight demographic characteristics with respect to size of the clusters using the combined genetic distance and participant high-risk contacts [11,24]. To identify the closest genetic links within each cluster, we used a python library, NetworkX, to construct a forest of minimum spanning trees (MST), which, like phylogeny, describes a network with the minimum number of genetic links that maintain connections between all cluster members [25]. We then compared genetic links found in the MST forest to those that were not selected by the algorithm to identify qualitative and quantitative differences.

The data analyzed in this article were collected and analyzed as part of CDC routine surveillance activities reported by multiple state and local health departments. This analysis was conducted only by CDC employees and contractors. CDC is not permitted to share or distribute any surveillance data due to an Assurance of Confidentiality authorized under Section 308 (d) of the Public Health Service Act (USA). Therefore, these data cannot be made publicly available by the authors. Each state has primary authority for determining whether their laws and regulations permit data submission to GenBank or other open databases. State and local health departments also have ability to determine whether and when these data can be shared with other researchers on a case-by-case basis.

## 3. Results

A total of 1326 STOP study participants were diagnosed with HIV-infection across three jurisdictions (North Carolina (NC) = 16.1%, New York City (NYC) = 50.8%, San Francisco (SF) = 33.1%) between 2011 and 2013. A majority (94.8%) were male, of non-white race (65.0%), with a median age of 30 years (interquartile range (IQR) 25.0–38.0) (Table 1). In aggregate, study participants had 4.6 anonymous partners for every named partner. After excluding those participants with incomplete interview responses (44.1%, *n* = 585), participants who met partners online (26.9%, *n* = 356) were 3.1 years (y) younger, claimed ≥4.0 (mean = 8.6) more total partners and named ≥1.2 (mean = 2.3) more high-risk contacts for partner notification than those that did not (29.0%, *n* = 385).

The recency status of HIV infection was determined for all study participants (*n* = 1326) and assumed established infection for all previously diagnosed partners. AHI was diagnosed in 12.6% (*n* = 167) of study participants and an established infection was diagnosed in the remaining 87.4% (*n* = 1159) participants. Partial HIV-1 *pol* sequences were obtained for 41.9% (*n* = 70) of participants diagnosed with AHI. In contrast, partial *pol* sequences were available for 41.3% (*n* = 547) of all study participants, thus, sequence availability was not biased. Among the available partial *pol* sequences, 32.9% (*n* = 180) had mutations that confer resistance to protease inhibitors (PIs), nucleoside reverse transcriptase inhibitors (NRTIs), or non-nucleoside reverse transcriptase inhibitors (NNRTIs). Further, 4.8% (*n* = 26) were also resistant to more than one class of antiretrovirals (ARVs); one person’s sequence showed resistance to all three classes of ARVs. Drug resistance associated mutations (DRAMs) were more prevalent in SF (33.2%) than in NYC (21.8%) or NC (22.4%) (*p* < 0.05). Minimal HIV-1 subtype diversity was observed (subtype B = 95.8%, *n* = 524). Participants with non-B subtypes were most prevalent in NYC (*n* = 11, 2.0%), followed by NC (*n* = 7, 1.3%) and SF (*n* = 5, 9.1%). Non-B subtypes consisted of CRF02_AG (*n* = 6, 1.1%), CRF01_AE (*n* = 3, 0.5%), CRF24_BG (*n* = 3, 0.5%), A1 (*n* = 3, 0.5%), C (*n* = 2, 0.4%), CRF12_BF (*n* = 2, 0.4%), D (*n* = 1, 0.2%), BC (*n* = 1, 0.2%), CRF44_BF (*n* = 1, 0.2%) and CRF06_cpx (*n* = 1, 0.2%).

Among 547 participants with newly diagnosed HIV with available *pol* sequences, 465 high-risk contacts were reported (epi links), of whom 35 (7.5%) had *pol* sequences available. Among these 35 HIV epi links, 23 (65.7%) were genetically supported and 12 (34.3%) were not genetically supported. Only five epi links (1.1%) were between participants with AHI, and none were genetically supported (Figure 1A). In contrast, phylodynamic inference identified 102 unreported putative transmission links, involving 97 unique participants. Among these unreported putative links, 12 (11.2%) were between persons with AHI, involving nine unique participants. The mean genetic distance between *pol* sequences from persons with AHI (d = 0.7%) was significantly lower than that of inferred links between persons with established HIV infections (d = 0.9%, *p* < 0.001), likely reflecting less intra-host evolution and, thus, more recent transmission between persons with AHI (Figure 1B).

We next compared the genetic distance (*d*) distributions (0 < *d* < 1.5%) for each category of putative transmission link between participants. There were no epi links between persons with AHI that were also supported by genetic evidence, but the distribution of Acute–Acute links is bimodal, with most occurring either below a genetic distance of 0.5% or above 1.0% (Figure 1B). Most of the genetic links between persons with established HIV infection (Established–Established) that were supported by an epi link fell below 0.5% (*n* = 13/23, 59.1%), whereas those without epi support were beyond 1.0% (*n* = 58/73, 79.5%). It is important to note that, barring mixed infections, the duration of AHI is insufficient for HIV to accrue sufficient genetic changes to achieve such distances > 1.0%. While the study was conducted over a three-year period, under normal circumstances this period remains insufficient to accrue such genetic distances between two acute cases. Therefore, links between persons with AHI where *d* > 1.0% are not likely to represent direct transmissions (*n* = 3/12, 25.0%), suggesting the presence of unobserved transmission intermediaries that might be elucidated by more robust contact tracing efforts. In contrast, close genetic links between persons with a supporting epidemiologic report are more likely to represent direct transmission partners. All putative transmission links between persons with AHI exhibited evidence of transmitted DRAM, where DRAMs were symmetrically distributed across each close genetic link. Notably, all putative transmission links between persons with acute HIV infection were unsupported by partner elicitation and occurred among large clusters (size ≥ 5).

**Figure 2:**
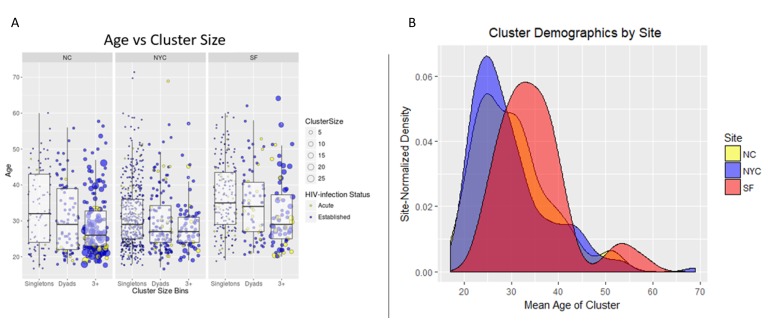
Age characteristics of cluster members, stratified by cluster size and study site (NC, SF, NYC). HIV transmission clusters varied with respect to size and the age of cluster members at the three study sites (North Carolina (NC), New York City (NYC), San Francisco (SF)). (**A**) Box-and-whisker plots of participant age, stratified by cluster size categories (singletons (*n* = 1), dyads (*n* = 2), and ≥3 (3+)) and study site. Underplotted behind the box-and-whisker plots are circles representing each participant, where the y-axis position corresponds to their age and circle size corresponds to cluster size. Each circle is colored by the recency of HIV-infection (Acute or Established) and scaled according to the size of their respective transmission clusters. (**B**) Kernel density plots showing the average age for participants of each cluster on the x-axis, colored by study site. Please note that the NC plot is behind the SF and NYC plots and does not always appear yellow in color in overlapping plot areas.

**Figure 3:**
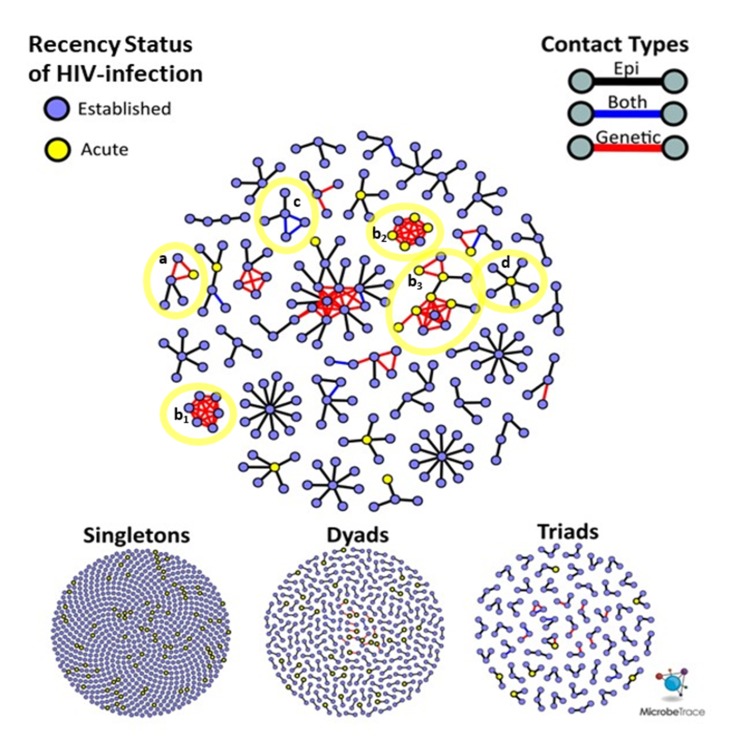
Superimposed risk networks and genetic clusters in three HIV study sites (North Carolina, San Francisco and New York City, stratified by cluster size. Network of all sexual contacts reported between participants and their partners diagnosed with HIV visualized using MicrobeTrace (http://microbetrace.cdc.gov); uninfected partners are not shown. A force-directed layout algorithm was applied to sort clusters by increasing size. Links are colored by contact type, ‘genetic only’ links in red, ‘epi only’ links in black and ‘both’ genetic and epi links in blue. Individuals are represented as circles, colored by recency of HIV infection. Blue circles represent established HIV-infection and yellow circles represent acute HIV-infection (AHI). Select clusters of interest are highlighted in yellow and labeled: Cluster (a) contains members in San Francisco and New York City. Clusters (b_1_), (b_2_) and (b_3_) consist primarily of members that did not name any high-risk contacts, where the majority of members of clusters (b_2_) and (b_3_) were diagnosed with acute HIV infection. Cluster (c) membership consists of two females who named each other as contacts and whose viruses are linked by a close genetic distance (*d* ≤ 1.5%).

The distribution of sexual network sizes varied across sampling sites, with small components of singletons (size = 1) and dyads (size = 2) most frequently found in SF and NYC (Table 2). Overall, as cluster size increased, the mean age of cluster members decreased (Figure 2A). Each site had bi-modal distributions of mean cluster age, where the second peak occurred at ≥50 years (Figure 2A,B). Notably, ages at individual- and cluster-levels in SF were right-shifted by approximately 10 years, indicating an older participant population, regardless of cluster size (Figure 2B). Each site had a single high-risk sexual network consisting of >10 members, but NC had the most sexual networks that consisted of at least five members (Figure 3). The largest network in NC (*n* = 26) was more than twice the size of the largest networks across all sites. Notably, all members of the two largest (size ≥ 5) SF networks exhibited transmitted drug resistance (TDR) and named zero high-risk partners. Two networks consisting of 2- and 4-members were inter-site, with members from NYC and SF. Inter-jurisdictional networks, dyads or larger networks representing more than one US state (*n* = 221, 77.5%) were three times more common than clusters involving only a single US jurisdiction.

Given that this study was conducted over three years, the recency status of HIV infection cannot be used to infer the order of transmission events. It is possible for nodes representing AHI to have joined a cluster prior to a node representing an established HIV infection. For example, an AHI node can reside at the center of a ‘star’-like cluster (Figure 3, notation D), surrounded by persons with established HIV infection who were interviewed after the acute index case.

Age and behavioral risk factors were the most powerful discriminators of molecular clustering among participants. Among the 547 participants with available *pol* sequences, those whose sequences clustered (*n* = 120) were younger than those that did not (*n* = 427) (*p* < 0.02). Unclustered participants differed most profoundly with respect to age from participants found in clusters of size ≥ 3, where the mean age disparity was 4.9 years (31.4 and 26.5 years, respectively) (*p* < 0.01). A similar difference (3.8 years) was observed between participants found in dyads (cluster size = 2) versus clusters of size ≥ 3 (30.3 and 26.5 years, respectively) (*p* < 0.03). The participants’ race had no effect on whether their *pol* sequence clustered with others.

**Figure 4:**
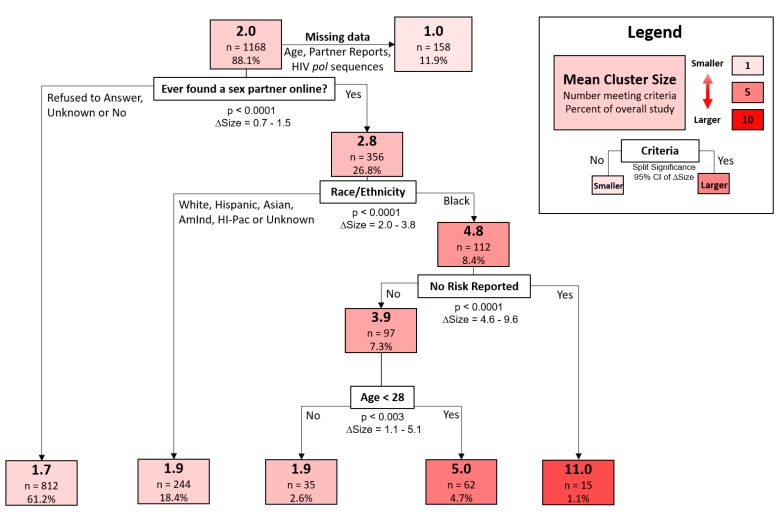
An example decision tree from the random forest model, trained to differentiate participants by cluster size based on their demographic and behavioral risk characteristics. Each colored box represents the number of participants meeting preceding criteria. Each box displays the mean cluster size, number of participants and their overall prevalence in the data. The criteria, statistical significance and confidence intervals of the difference in mean cluster size (∆Size) are displayed below each branching point of the tree.

A decision-tree model was used to classify cluster sizes according to participant demographic and behavioral traits. The strongest discriminator of increased cluster size, representing the first split of the decision tree, was whether a participant reporting meeting sexual partners online (95% confidence interval of size difference [CISD] = 0.7–1.5, *p* < 0.0001) (Figure 4). Black participants who met partners online were found in larger clusters (mean size = 4.8, *n* = 112) than all other races that also met partners online (mean size = 1.9, *n* = 244) (95% CISD = 2.0–3.8, *p* < 0.0001). Those found among the largest clusters were black participants who met sexual partners online but reported no risk factors during the interview (mean size = 11.0, *n* = 15). In contrast, black individuals who met partners online but did report risk factors were in substantially smaller clusters (mean size = 3.9, *n* = 97) (95% CISD = 4.6–9.6, *p* < 0.0001). Finally, young black participants (age < 28, mean size = 5.0, *n* = 62) that reported high-risk behavior and meeting partners online were found in larger clusters than their older counterparts (age > 28, mean size = 1.9, *n* = 35) (95% CISD = 1.1–5.1, *p* < 0.003).

## 4. Discussion

In this study of persons with newly diagnosed HIV infection, phylodynamics analyses provided important complementary information to epidemiologic data obtained by traditional partner services. Implementation of a more holistic public health approach can be leveraged to rapidly detect and respond to emerging clusters of HIV infection, which is a strategic pillar of the proposed initiative, *Ending the HIV Epidemic*, *A Plan for America* [26]. Phylodynamics demonstrated that approximately one-third of named partners with newly diagnosed HIV infection had a viral strain that was too genetically distant from the study participant to represent an actual HIV transmission. Almost 20% of study participants had a viral strain that molecularly clustered with another study participant without any epidemiologic evidence of linkage. Decision-tree analysis of partner services data indicated a high number of anonymous sexual partners. The increased use of the internet to meet sexual partners likely contributed to these findings. Although phylodynamics identified hidden transmission linkages, these analyses cannot replace traditional partner services efforts as they do not provide direct outreach to persons at the highest risk for HIV infection. Conversely, they do demonstrate the limitations of partner services alone to understand and implement prevention measures to interrupt on-going HIV transmission. Indeed, the lack of agreement between these methods suggests that molecular techniques can augment traditional methods by addressing gaps that result from anonymous partners, imperfect reporting or an overburdened epidemiologic workforce [9,12,27,28].

We characterized the distribution of close genetic links between all pairs of HIV *pol* sequences with respect to the recency of HIV infection, presence of symmetrical drug-resistance mutations and existence of a corresponding report of high-risk contact. We also characterized links according to demographic, geographic and behavioral concordance. Where sufficient data were available, the integration of bioinformatics and laboratory methods with partner services interviews provided a quantitative means of assessing linkage by direct or indirect transmission. Our results also highlight the demographic and behavioral composition of transmission clusters in both similar and disparate geographic and socioeconomic environments. We found that molecular epidemiology and traditional partner services investigations offer complementary perspectives on high-risk behaviors associated with transmission of infectious diseases across high-risk contact networks. Perhaps more importantly, enhanced partner services did not identify forward transmission among persons with acute HIV infection. In contrast, a molecular clustering approach identified evidence of forward transmission of drug resistant HIV among persons with AHI in more than one large (size ≥ 5) cluster. Indeed, all close genetic links between persons with AHI were found among large clusters. In light of (1) high viral loads maintained during the acute phase of infection, (2) the inherent flaws in partner elicitation [12,27,28], and (3) observations that persons with AHI name genetic partners with increased likelihood [12]; genetic linkage (*d* ≤ 1.5%) between persons with AHI – especially those that do not name each other as contacts – may represent the presence of unobserved or undiagnosed intermediaries. This is increasingly likely when the genetic distance between partial *pol* sequences exceeds 1.0% (*d* > 1.0%). These results were robust to moderate variations in the HIV genetic distances, known to accrue over spans of ≤5 years, due in part to supporting laboratory and epidemiologic data.

The average combined cluster size, which included named partners who had prior knowledge of their HIV-infected status and were therefore not included in the study, varied markedly by site. Differences in the geographic scale, type and availability of services, and variation in demographics or population density could each contribute to the different trends in cluster sizes observed between North Carolina and more urban San Francisco and New York City sites. Similarity between San Francisco and New York City was supported by the presence of multiple clusters with members in both cities. Upon completion of partner services investigations of the largest cluster, observed in North Carolina, we found the cluster consisted of six non-overlapping sexual networks that were later linked by molecular evidence as a 25-member cluster consisting of mostly Black Men Who Have Sex With Men (MSM) While an outlier, this cluster’s size is instructive as it may reflect an environment in which affected communities are more insular. Black MSM are a small subset of all MSM, and their partners tend to be of the same race. Because of the small population size and higher prevalence of HIV in that population relative to other races/ethnicities, Black MSM are at greater risk of being exposed to HIV within their sexual networks as has been reported in numerous studies, including larger studies in North Carolina. Recently, other studies have described multiple sexual HIV transmission clusters within NYC and NC by integrating partner notification networks and either phylogenetics or network analyses [12,27,28]. Concordant with our findings, these studies also demonstrated the utility of incorporating both networks for identifying hidden HIV transmission and facilitating targeted prevention strategies to clusters found to be rapidly growing with new infections.

High-risk behaviors and youth were generally strong decision-tree predictors of genetic clustering and the overall size of the sexual networks in which a participant was observed. For example, having met a sexual partner online was identified by random forest analysis to be the strongest discriminator of small and large clusters. The random forest analysis indicates that those participants found among the largest clusters were Black men who reported meeting partners online but reported no risk factors, suggesting the important role of non-disclosure of MSM as a risk factor for HIV infection [29]. The discrepancy between the numbers of named and claimed high-risk partners, taken to be the number of anonymous partners herein, was also associated with large clusters. When cluster size is treated as a categorical variable, rather than a continuous one, we find that larger clusters are typically associated with younger participants irrespective of site. The age ranges and stratification among singletons, dyads and clusters ≥ 3 in size were inconsistent across sites, which suggests differing sociodemographic environments and the need for different prevention strategies for different HIV clusters.

In summary, we evaluated each type of link between participants by epidemiologic and HIV genetic distance. One third of participants with HIV sequences who were linked epidemiologically were not genetically linked as transmission partners, highlighting the high rate of false positive linkage by enhanced partner services. Genetic linkage, and not enhanced partner services, identified rapidly growing clusters composed of participants with AHI, further supporting the importance of integrating molecular clustering techniques with partner services to prioritize prevention responses.

## Figures and Tables

**Table 1:** Demographic characteristics of clustered and unclustered HIV sequences in NYC, SF and NC.

Characteristic	Clustered (*n* = 120)	Unclustered (*n* = 427)	Clustered OR * [95% CI ^#^]	*p*-Value
**Site**				
New York City (NYC)	37 (18.3%)	165 (81.7%)	1.07 [0.62–1.83]	
San Francisco (SF)	55 (29.9%)	129 (70.1%)	2.03 [1.21–3.40]	
North Carolina (NC)	28 (17.4%)	133 (82.6%)	Ref ^$^	
**Mean Age**				
	28.9	32.7	-	*p* < 0.001
**Men who have sex with men**				
Yes	98 (22.8%)	331 (77.2%)	0.68 [0.26–1.83]	
No	15 (20.8%)	57 (79.2%)	Ref	
Unknown	7 (15.2%)	39 (84.8%)	1.13 [0.61–2.07]	
**Gender**				
Male	113 (22.6%)	388 (77.4%)	Ref	
Female	4 (16.0%)	21 (84.0%)	0.65 [0.22–1.95]	
Other	3 (14.3%)	18 (85.7%)	1.15 [0.23–5.75]	
**Race/ethnicity**				
Black	35 (17.5%)	165 (82.5%)	0.72 [0.43–1.19]	
Hispanic	24 (24.2%)	75 (75.8%)	1.08 [0.61–1.93]	
White	40 (22.9%)	135 (77.1%)	Ref	
Other	21 (28.8%)	52 (71.2%)	2.41 [1.14–5.11]	
**HIV Infection Status**				
Acute (AHI)	24 (20.3%)	94 (79.7%)	0.88 [0.53–1.46]	
Established	96 (22.3%)	333 (77.6%)	Ref	
**Drug Resistance Associated Mutations (DRAMs)**				
	37 (26.2%)	104 (73.8%)	1.39 [0.89–2.16]	
**Partners**				
Mean ^#^ of named partners	2.01	1.66	-	*p* = 0.21
Mean ^#^ of anonymous partners	4.6	3.7	-	*p* = 0.76
Mean ^#^ of AHI named or molecularly linked partners	0.69	0.04	-	*p* < 0.001
Mean ^#^ of named or molecularly linked partners with established infection	2.35	0.50	-	*p* < 0.001

OR *—Odds Ratio. CI ^#^ —Confidence Interval. Ref ^$^ —Reference group.

**Table 2:** Distribution of HIV cluster sizes by study site (NC, SF, NYC).

Site	Cluster Size *n* = 1	Cluster Size *n* = 2	Cluster Size *n* = 3	Cluster Size *n* = 4	Cluster Size *n* ≥ 5
North Carolina (NC)	128 (9.7%)	32 (2.4%)	21 (1.6%)	4 (0.3%)	27 (2.0%)
San Francisco (SF)	366 (27.6%)	33 (2.5%) *	6 (0.5%)	1 (0.1%) *	33 (2.5%)
New York City (NYC)	593 (44.7%)	41 (3.1%) *	21 (1.6%)	7 (0.7%) *	11 (0.8%)

* Counts include members of inter-site clusters.
